# Claudin‐3 Deficiency Potentially Permits Epidermal Nerve Infiltration and Contributes to Sensitive Skin Pathophysiology

**DOI:** 10.1111/1346-8138.70403

**Published:** 2026-07-26

**Authors:** Arisa Kato, Akane Kawamoto, Daiki Murase, Hiroyuki Yoshida, Kenji Kabashima

**Affiliations:** ^1^ Skin Beauty 1 Products Research, Kao Corporation Odawara Japan; ^2^ Department of Dermatology Kyoto University Graduate School of Medicine Kyoto Japan

**Keywords:** claudin‐3, epidermal nerve fiber, epidermis, keratinocytes, sensitive skin, tight junction

Sensitive skin (SS) is a clinical condition characterized by unpleasant sensory reactions—such as itching, burning, or stinging—triggered by various environmental factors [1]. Key clinical characteristics of SS include altered trans‐epidermal water loss (TEWL), disrupted ceramide profiles, and reduced thresholds to stimuli [1, 2, 3]. While both epidermal barrier dysfunction and neurosensory dysregulation are considered contributors to SS, its neuroanatomical basis remains poorly understood, largely due to limited histopathological studies of SS skin.

Recently, epidermal nerves were reported to be contained underneath tight junctions (TJs) formed in the epidermal granular layer and protected from overexposure to environmental stimuli in normal human skin [4]. In the present study, we aimed to explore the relationship between epidermal nerve ending localization and cutaneous hypersensitivity by focusing on the granular layer and its potential mechanistic underpinnings, using skin biopsies obtained from individuals with SS and non‐sensitive skin (NS).

We classified the individuals as NS or SS using a combined assessment of a self‐rating questionnaire and 10% lactic acid stinging test (LAST) (Supporting Information Materials and Methods). The three participants classified as SS were the top three scorers on LAST, with a mean score of 1.83 ± 0.29 (mean ± SD), suggesting mild‐to‐moderate skin hypersensitivity. Compared with NS individuals, those with SS tended to have higher TEWL (*p* = 0.057) with no significant differences in capacitance, indicating subtle barrier impairment despite the absence of visible abnormalities (Table S1).

To first investigate whether anatomical changes in epidermal nerves are present in SS individuals, we performed immunohistochemical staining of skin tissues obtained from NS and SS individuals for a pan‐neuronal marker PGP9.5. As shown in Figure 1a, the majority of PGP9.5^+^ nerve fibers were detected in the middle layer of the epidermis in both SS and NS individuals. However, SS samples exhibited a notably higher number of fibers extending upward through claudin‐4‐positive regions, which span from the spinous to granular layers [5], to just beneath the stratum corneum (SC). Quantitative analysis confirmed that the number of fibers approaching the SC was significantly higher in SS skin (Figure 1b). Because epidermal nerve endings were reported to be pruned to stay below the TJs, and disruption of this can result in aberrant epidermal nerve activation [4], we hypothesized that TJ dysfunction may contribute to the abnormal localization of sensory nerve endings.

**FIGURE 1:**
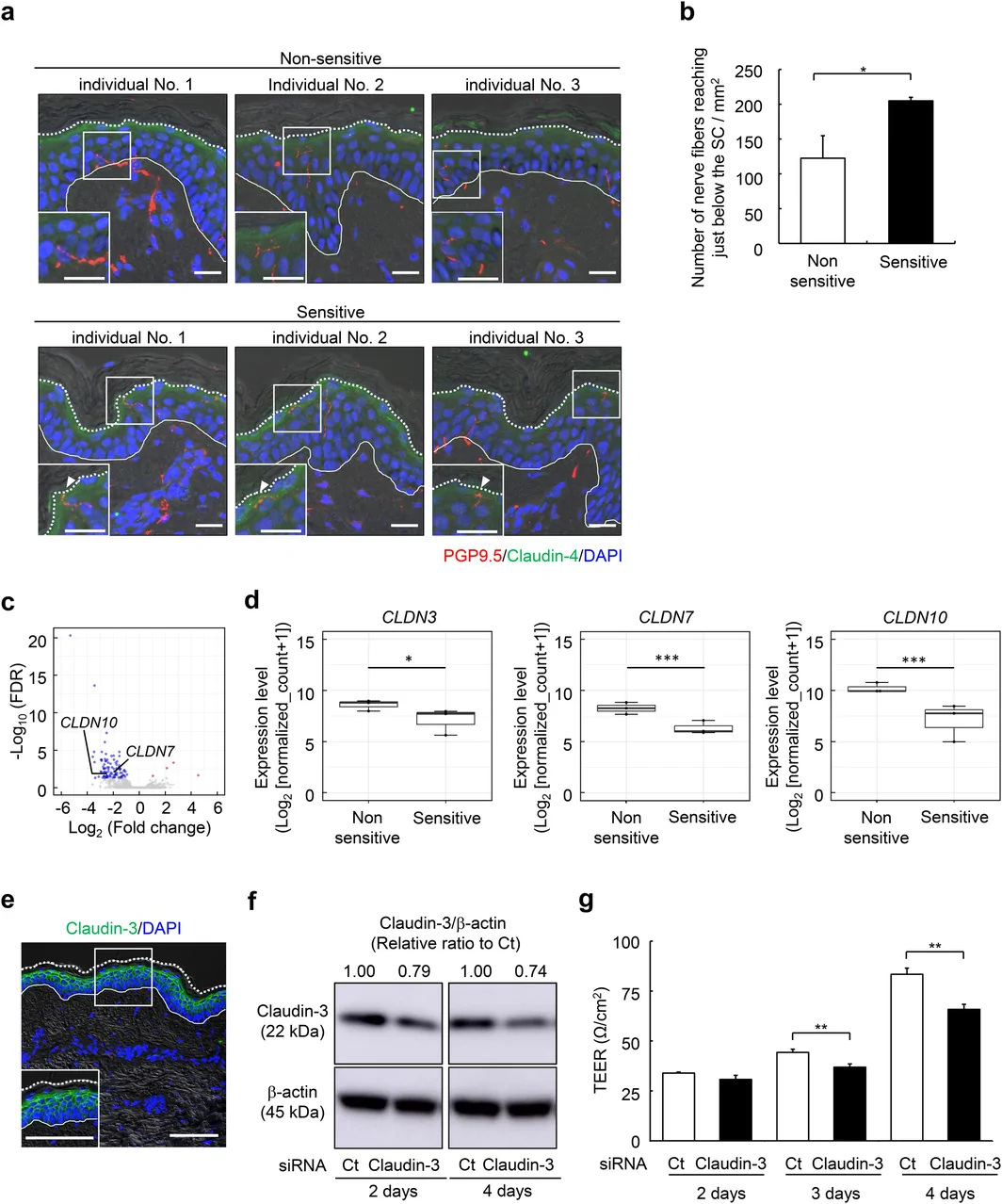
Aberrant localization of epidermal nerve endings in sensitive skin tissues accompanied by differential gene expression patterns, highlighting essential contribution of claudin‐3 to regulating tight junctions in keratinocytes. Skin biopsies were taken from individuals with SS (*n* = 3) and NS (*n* = 3). (a) Double‐immunostaining for PGP9.5 (*red*) and claudin‐4 (*green*) merged with DAPI (*blue*). Arrowheads indicate PGP9.5^+^ nerve fibers reaching just below the stratum corneum (SC). Scale bars = 20 μm. Insets, high‐power view of boxed areas. Dotted lines indicate the bottom of the SC and solid lines mark the epidermis‐dermis border. (b) The numbers of PGP9.5^+^ nerve fibers reaching just below the SC were normalized by the epidermal area. The values represent mean ± SD, *n* = 3. *p < 0.05 (Student's *t*‐test). (c) Volcano plot of genes (*red*, upregulated; *blue*, downregulated) in SS and NS. Benjamini and Hochberg's false discovery rate (FDR) < 0.05 (*n* = 3). (d) Claudin genes expression in SS and NS. Boxes represent mean ± interquartile range (IQR), and whiskers represent 1st and 3rd quartile 1.5× IQR. ***p < 0.001; *p < 0.05 (Wald test, *n* = 3). (e) Immunostaining for claudin‐3 (*green*). Scale bar = 100 μm. Dotted line, SC surface. Solid line, the epidermis‐dermis border. (f) Immunoblotting for claudin‐3 in keratinocytes treated with claudin‐3 or control (Ct) siRNA. The number indicates claudin‐3 expression normalized to β‐actin as a relative ratio to Ct. (g) Transepithelial electrical resistance (TEER) in claudin‐3 siRNA‐treated keratinocytes (*n* = 3). **p < 0.01 (Student's *t*‐test).

To test this, we performed transcriptomic analysis of skin tissues and identified 4 upregulated and 93 downregulated genes (FDR < 0.05) in SS individuals compared with NS individuals (Figure 1c). Among these targets, mRNA levels of TJ components *claudin* (*CLDN*) *7* and *CLDN10* were significantly decreased in SS individuals than those in NS individuals (Figure 1d). The mRNA level of *CLDN3*, known as a human epidermal TJ gene [6], was significantly decreased in SS individuals (*p* < 0.05) (Figure 1d).

Immunohistochemical analysis showed claudin‐3 localized from suprabasal to granular layers (Figures 1e and S1), implying a fundamental role of TJ formation. Meanwhile, claudin‐7 and ‐10 were not detected in the whole epidermis (data not shown). Although quantitative claudin‐3 signals did not differ significantly between SS and NS individuals—likely because the assay lacked sensitivity to detect the subtle mRNA‐level changes—reduced *CLDN3* expression may contribute to the abnormal nerve localization in SS skin.

To our knowledge, there is no direct evidence showing that claudin‐3 plays a functional role in epidermal TJs. We therefore assessed the impact of claudin‐3 knockdown using siRNA on transepithelial electrical resistance (TEER), a measure of TJ barrier integrity, in cultured normal human epidermal keratinocytes (NHEKs). As shown in Figure 1f,g, siRNA‐targeted knockdown of claudin‐3 significantly reduced TEER in NHEKs, indicating that claudin‐3 is a functional determinant of epidermal TJs. Notably, even a mild reduction in claudin‐3 expression—as observed in SS individuals—caused a marked TEER decline, suggesting a high sensitivity of TJ function to claudin‐3 levels. Given that CLDN3 functions as a sealing claudin contributing to epithelial barrier integrity [7], reduced claudin‐3 expression in SS may impair epidermal TJ function, allowing nerve endings to extend toward the skin surface and contribute to cutaneous hypersensitivity.

In conclusion, by focusing on epidermal barrier–sensory nerve interactions in the granular layer, this study provides preliminary evidence that epidermal nerves intruding just beneath the SC may underlie cutaneous hypersensitivity in individuals with SS. Moreover, we identify claudin‐3 as a novel functional keratinocyte TJs component whose slight, consistent reduction may drive this aberrant nerve localization observed in SS. Limitations include a small sample size and restrictions in anatomical site, age range, gender, and ethnicity; larger, more diverse studies are needed. Despite these limitations, our findings offer novel perspectives on the pathophysiology of SS and highlight the importance of epidermal nerve–TJ interactions in maintaining cutaneous sensory homeostasis.

## Ethics Statement

This study was conducted in accordance with the Declaration of Helsinki and approved by the Institutional Review Board of Tokyo Sta. Center Building Clinic (Tokyo, Japan) (Clinical Trial Protocol number: ODW20210126) and the Ethical Committee of Kao Corporation (Approval number: D167‐210215). Participants provided written informed consent.

## Conflicts of Interest

The authors declare no conflicts of interest.

## Supporting information


**Figure S1:** Localization of claudin‐3 and claudin‐4 in skin tissue. Skin biopsies were taken from individuals with NS. Double immunostaining for claudin‐4 (red) and claudin‐3 (green) merged with DAPI (blue). Scale bar = 50 μm. Dotted line, SC surface. Solid line, the epidermis‐dermis border.

**Data S1:** Supporting Information Materials and Methods.

**Table S1:** Clinical characteristics of the study individuals.

## Data Availability

Data related to this article are available from the corresponding authors upon reasonable request.

## References

[1] L. Misery , K. Loser , and S. Ständer , “Sensitive Skin,” Journal of the European Academy of Dermatology and Venereology 30, no. suppl. 1 (2016): 2–8, 10.1111/jdv.13532. 26805416

[2] M. A. Farage , “The Prevalence of Sensitive Skin,” Frontiers in Medicine (Lausanne) 6 (2019): 98, 10.3389/fmed.2019.00098. PMC6533878 31157225

[3] T. Joichi , H. Yoshida , H. Katsukura , et al., “Altered Ceramide Profile of Facial Sensitive Skin: Disordered Intercellular Lipid Structure Is Linked to Skin Hypersensitivity,” Journal of Cosmetic Dermatology 24, no. 4 (2025): e70154, 10.1111/jocd.70154. 40176380 PMC11965967

[4] S. Takahashi , A. Ishida , A. Kubo , et al., “Homeostatic Pruning and Activity of Epidermal Nerves Are Dysregulated in Barrier‐Impaired Skin During Chronic Itch Development,” Scientific Reports 9, no. 1 (2019): 8625, 10.1038/s41598-019-44866-0. 31197234 PMC6565750

[5] T. Yuki , M. Tobiishi , A. Kusaka‐Kikushima , Y. Ota , and Y. Tokura , “Impaired Tight Junctions in Atopic Dermatitis Skin and in a Skin‐Equivalent Model Treated With Interleukin‐17,” PLoS One 11, no. 9 (2016): e0161759, 10.1371/journal.pone.0161759. 27588419 PMC5010286

[6] J. M. Brandner , M. Zorn‐Kruppa , T. Yoshida , I. Moll , L. A. Beck , and A. De Benedetto , “Epidermal Tight Junctions in Health and Disease,” Tissue Barriers 3, no. 1–2 (2015): e974451, 10.4161/21688370.2014.974451. 25838981 PMC4372028

[7] S. Milatz , S. M. Krug , R. Rosenthal , et al., “Claudin‐3 Acts as a Sealing Component of the Tight Junction for Ions of Either Charge and Uncharged Solutes,” Biochimica et Biophysica Acta—Biomembranes 1798, no. 11 (2010): 2048–2057, 10.1016/j.bbamem.2010.07.014. 20655293

