# Supplementary material for: Claudin‐3 Deficiency Potentially Permits Epidermal Nerve Infiltration and Contributes to Sensitive Skin Pathophysiology
Source: J Dermatol. 2026 Jul 26;53(9):1392–4. doi: 10.1111/1346-8138.70403 (PMC13555158; doi:10.1111/1346-8138.70403)
Supplement: Supplementary file 1 [file JDE-53-1392-s003.pdf]

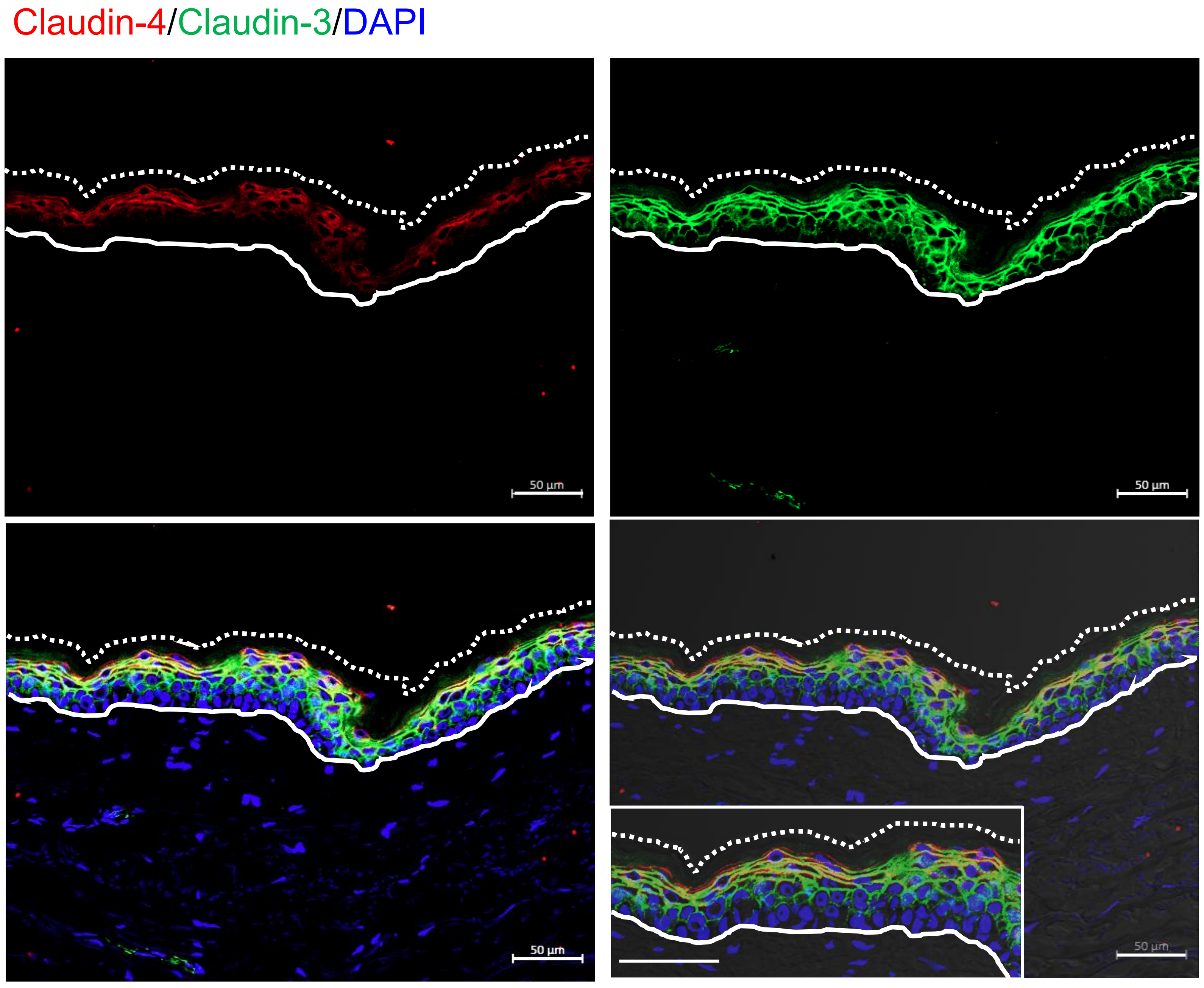

Supplementary Figure 1. Localization of claudin-3 and claudin-4 in skin tissue. Skin biopsies were taken from individuals with NS. Double-immunostaining for claudin-4 (*red*) and claudin-3 (*green*) merged with DAPI (*blue*). Scale bar = 50 µm. Dotted line, SC surface. Solid line, the epidermis-dermis border.
