# Supplementary material for: Claudin‐3 Deficiency Potentially Permits Epidermal Nerve Infiltration and Contributes to Sensitive Skin Pathophysiology
Source: J Dermatol. 2026 Jul 26;53(9):1392–4. doi: 10.1111/1346-8138.70403 (PMC13555158; doi:10.1111/1346-8138.70403)
Supplement: Supplementary file 2 [file JDE-53-1392-s001.docx]

**SUPPLEMENTARY MATERIALS AND METHODS**

**Study individuals and skin biopsy processing**

For this study, a screening pool of 114 healthy female individuals (age range, 20–59 years) was evaluated by self-rating questionnaire on skin hypersensitivity and a 10 % lactic acid stinging test (LAST) as described below. Individuals with visible skin disease or significant medical conditions were excluded.

Based on the self-rating questionnaire, 32 individuals who self-identified as having sensitive skin (SS) and 11 individuals who self-identified as having non-sensitive skin (NS) were identified. For biopsy, three SS individuals with LAST scores ≥ 1.5 (top three score values; mean age ± SD, 47.67 ± 4.79 years) and three NS individuals selected from those with LAST scores of 0 (n = 4) considering the age difference relative to the SS group (mean age ± SD, 43.00 ± 7.68 years) were enrolled, and skin biopsy was subsequently performed from the upper arm where LAST was conducted.

The collection of skin samples was performed at Yaesu Nihonbashi Skin Clinic (Tokyo, Japan). After the biopsy, each skin tissue was divided into two equal pieces. Half of the tissue was immediately embedded in the Tissue-Tek optimal cutting temperature compound (Sakura Finetek, Tokyo, Japan) for immunohistochemical analysis with frozen skin sections. The other half of the skin tissue was immediately fixed in 4% paraformaldehyde (FUJIFILM Wako Pure Chemical Corporation, Osaka, Japan) at 4°C and washed in phosphate-buffered saline (PBS) and embedded in paraffin.

**Questionnaire for self-diagnosis of SS**

Based on their responses to the self-rating questionnaire concerning hypersensitivity of body skin, the individuals were enrolled in the self-perceived SS group or the self-perceived NS group. The questionnaire comprises three questions: whether you aware of having sensitive skin on the body, you had experienced stinging or burning sensations after using cosmetics, and you described your skin as easily irritated and pone to redness.

**LAST**

The solution of 10% aqueous lactic acid (Musashino lactic acid 90, Musashino Chemical Laboratory, Tokyo, Japan) and distilled water as a control were applied to the upper arm. The sensory irritation score was respectively recorded at 3 specific time points (30 sec, 2.5 min, and 5 min) with 7 grades as follows: 0, no sensation; 0.5, slight discomfort, barely detectable; 1.0, mild discomfort; 1.5, mild to moderate discomfort; 2.0, moderate discomfort; 2.5, moderate to severe discomfort; and 3.0, severe discomfort. The LAST score was calculated by subtracting the score value for distilled water application from that for 10% aqueous lactic acid application. The individuals with self-perceived SS and LAST scores ≥ 1.5 were classified as SS, otherwise the individuals with self-perceived NS and LAST score = 0 were classified as NS.

**Assessment of epidermal barrier function (skin capacitance and trans-epidermal water loss [TEWL])**

Each participant was acclimated in a room at 23 ± 2°C with constant humidity (45 ± 5%) for at least 20 min prior to the measurements. The stratum corneum (SC) capacitance was measured with Corneometer CM825 (Courage & Khazaka, Electronic, Cologne, Germany), and TEWL was measured using Tewameter TM 300 (Courage & Khazaka, Electronic). All measurements were conducted on the upper arm before the skin biopsy was performed.

**RNA-sequence (RNA-seq) analysis**

To analyze the transcriptome of the whole skin tissues, frozen skin sections (20 μm thick) from the skin biopsy samples were mounted on membrane slides (PEN-Membrane 2.0　μm, Leica Microsystems, Wetzlar, Germany), then two frozen sections were directly collected into RLT buffer (Qiagen, Hilden, Germany) containing 40 μM dithiothreitol (Sigma-Aldrich, St. Louis, MO, USA). Total RNA was extracted and purified using the RNeasy kit (Qiagen). The preparation for sequence library, template and sequencing were performed using the Ion AmpliSeq Transcriptome Human Gene Expression Kit, Ion Chef System and Ion S5 XL System (Thermo Fisher Scientific, Waltham, MA, USA).

**RNA-seq data analysis and statistics**

Read count data were obtained using AmpliSeq RNA plug-in of the Ion Torrent Suite Software v.5.14.0 (Thermo Fisher Scientific). Read count normalization and all statistical analysis were performed with R language. The R DESeq2 package (Bioconductor) was used for normalization and quality check of read count data. We analyzed only genes with non-zero read counts in more than all samples, and these genes were normalized by a size factor. Differentially expressed genes　between the individuals with SS and NS were analyzed by Wald test, and those with a false discovery rate were adjusted by the Benjamini–Hochberg method.

**Immunofluorescence microscopic analysis**

For immunofluorescence of frozen tissues, skin sections on glass slides were fixed with methanol at -20°C for 10 min. The slides were blocked with 1% bovine serum albumin (BSA) in PBS, then incubated with primary antibody (rabbit anti-claudin 3 antibody and mouse anti-claudin 4 antibody; 1:200 dilution, Thermo Fisher Scientific) overnight at 4°C, followed by incubation with secondary antibody (anti-rabbit Alexa Fluor 488 and anti-mouse Alexa Fluor 647; 1:200 dilution, Thermo Fisher Scientific) at room temperature for 60 min. For immunofluorescence of formalin fixed paraffin embedded tissues, skin sections on glass slides were hydrated by passage through xylene and graded ethanol. After antigen retrieval for 15 min at 100°C in Tris-EDTA (ethylenediaminetetraacetic acid) buffer (pH 9.0), the slides were permeabilized in 0.2% Triton X-100 in PBS for 10 min at room temperature. The slides were blocked with 1% BSA in PBS, then incubated with primary antibody (rabbit anti-PGP9.5 antibody; 1:100 dilution, Abcam, Cambridge, UK; mouse anti-claudin-4 antibody; 1:100 dilution, Thermo Fisher Scientific) overnight at 4°C, followed by incubation with secondary antibody (anti-rabbit Alexa Fluor 647 and anti-mouse Alexa Fluor 488; 1:200 dilution, Thermo Fisher Scientific) at room temperature for 60 min. Imaging was performed using a Zeiss LSM710 confocal microscopy system (Carl Zeiss Microscopy GmbH, Jena, Germany). For analysis of localization of PGP9.5 positive (PGP9.5^+^) nerve fibers, Z-stack images were acquired as maximum-intensity projections using a ZEN software (Carl Zeiss Microscopy GmbH). Z-stack images covered a 7.04 μm thickness of skin sections with 0.88 μm interval between the individual images in a Z-stack. Representative images shown in this study are the maximum intensity projections of Z-stacks. The number of PGP9.5^+^ nerve fibers reaching just blow the SC were quantified using the image analysis software Image J (National Institutes of Health, Bethesda, MD, USA) and normalized by the epidermal area. Counting of nerve fibers were independently performed by three evaluators.

**Cell culture**

Normal human epidermal keratinocytes (NHEKs) (Kurabo, Osaka, Japan) were routinely cultured in HuMedia-KG2 medium supplemented with human keratinocyte growth supplement (Kurabo) in a humidified atmosphere with 5% CO_2_ at 37°C. Upon reaching confluence, cells were transferred to 1.8 mM Ca^2+^ medium and cultured for different periods before harvest.

**Small interfering RNA (siRNA) transfection**

For siRNA transfection, Lipofectamine RNAiMAX Transfection Reagent (ThermoFisher Scientific) and Opti-MEM medium (ThermoFisher Scientific) were used. NHEKs were transfected with 5 nM claudin-3 siRNA (ThermoFisher Scientific) or Silencer™ Select Negative Control No.1 siRNA (control siRNA, ThermoFisher Scientific), then the transfected cell suspension was seeded onto the upper side of transwell culture inserts (pore size 0.4 μm, Corning, Corning, NY, USA). Only culture media mixed with siRNA or control siRNA were added to the lower side of the transwell culture inserts. Cells were used for experiments 24 h after transfection.

**Transepithelial electrical resistance (TEER) evaluation**

The cellZscope tight junction monitoring system (CellSeed Inc, Tokyo, Japan) was used to measure the TEER of NHEKs seeded onto the transwell culture inserts (pore size 0.4 μm, Corning) in1.8 mM Ca^2+^ medium. The TEER was evaluated at 2, 3, and 4 days after the treatment with claudin-3 or control siRNAs.

**Immunoblotting**

Cell samples were solubilized at 4°C in RIPA Lysis Buffer (Sigma-Aldrich) containing protease/phosphatase inhibitor (Cell Signaling Technology, Danvers, MA, USA), then total protein was obtained from the supernatant followed by sonication and centrifugation. Total protein was separated on NuPAGE™ 12% Bis-Tristampak gels (ThermoFisher Scientific) and transferred onto polyvinylidene fluoride membranes (Merck, Germany, DE). Non-specific protein binding was blocked by incubating membranes in a solution of 5% skim milk (FUJIFILM Wako Pure Chemical Corporation) in Tris-buffered saline with Tween 20 (Cell Signaling Technology) for 60 min at room temperature. After blocking, the membranes were incubated with primary antibodies (rabbit anti-β-actin antibody; 1:2000 dilution, Cell Signaling Technology; rabbit anti-claudin-3 antibody; 1:2000 dilution, Thermo Fisher Scientific) overnight at 4°C, followed by incubation with horseradish peroxidase-conjugated secondary antibody (anti-rabbit antibody; 1:5000 dilution, Danaher Corporation, Washington DC, USA) for 60 min at room temperature. Protein bands were detected using ECL™ Prime Western Blotting Detection Reagent (Cytiva, Bucks, UK) and the intensity of the bands was quantified using an Amersham Imager 600 (GE Healthcare, Chicago, IL, USA).

**Statistical analysis**

Data were compared using Student's *t*-test, Mann-Whitney U-test, and Wald test. Statistical significance was set at *p* < 0.05 and a false discovery rate were adjusted by the Benjamini–Hochberg method below the threshold value of 0.05. All statistical analyses were performed using IBM SPSS Statistics 29.0.2.0 (IBM, Armonk, NY, USA), Microsoft Excel (Office 365) (Microsoft, Redmond, WA, USA) software, or R language.
