# Supplementary material for: Claudin‐3 Deficiency Potentially Permits Epidermal Nerve Infiltration and Contributes to Sensitive Skin Pathophysiology
Source: J Dermatol. 2026 Jul 26;53(9):1392–4. doi: 10.1111/1346-8138.70403 (PMC13555158; doi:10.1111/1346-8138.70403)
Supplement: Supplementary file 3 [file JDE-53-1392-s002.docx]

**Supplementary Table 1. Clinical characteristics of the study individuals.**

|  | Individuals with NS | Individuals with SS | *p*-value |
| --- | --- | --- | --- |
| LAST score | 0 | 1.83 ± 0.29 | 0.034* |
| Capacitance (A.U.) | 33.11 ± 16.83 | 41.17 ± 13.71 | N.S.  (0.555) |
| Trans-epidermal water loss (TEWL) (g/h/m²) | 10.10 ± 0.83 | 13.32 ± 1.93 | N.S.  (0.057) |

LAST score, capacitance, and TEWL of the individuals with sensitive skin (SS) (n=3) and non-SS (NS) (n=3). Values are expressed as mean ± standard deviation (SD). A.U., arbitrary unit. Statistical significance was assessed using Mann-Whitney U-test for LAST score and Student's *t* test for capacitance and TEWL values. *, Asymptotic significance *p*-values (two tailed-test) < 0.05; N.S., not significant.
